# MicroRNAs in Salivary Gland Cancers Associated with Poor Prognosis: A Systematic Review

**DOI:** 10.3390/ijms27146101

**Published:** 2026-07-08

**Authors:** Julia Pikul, Maja Cieślik, Kazimierz Niemczyk, Anna Rzepakowska

**Affiliations:** 1 Department of Otorhinolaryngology, Head and Neck Surgery, Medical University of Warsaw, 02-097 Warsaw, Poland; kazimierz.niemczyk@wum.edu.pl (K.N.); a.rzepakowska@wum.edu.pl (A.R.); 2 Department of Epidemiology and Biostatistics, Medical University of Warsaw, 02-091 Warsaw, Poland; maja.cieslik@wum.edu.pl; 3 Doctoral School, Medical University of Warsaw, 02-091 Warsaw, Poland

**Keywords:** salivary gland cancer, adenoid cystic carcinoma, mucoepidermoid carcinoma, miRNA, clinicopathological features, outcome, poor prognosis, biomarkers

## Abstract

Salivary gland cancers (SGCs) are rare and heterogeneous tumours, making the identification of reliable prognostic biomarkers challenging. MicroRNAs (miRNAs) regulate post-transcriptional gene expression and may play important roles in tumour progression and treatment response. This systematic review aims to identify and summarize the current evidence on miRNA dysregulation in SGCs, with a particular focus on their prognostic relevance and therapeutic potential. A systematic search of PubMed/MEDLINE, Embase, Web of Science, and the Cochrane Library was conducted up to 3 December 2025, following PRISMA guidelines and a pre-registered protocol (CRD420251231827). Observational cohort and cross-sectional studies reporting microRNA dysregulation in human salivary gland tissues with prognostic assessment were included. Risk of bias was assessed using the Quality in Prognosis Studies (QUIPS) tool. Due to considerable heterogeneity, a narrative synthesis was conducted. Twenty studies published between 2013 and 2025 that fulfilled the search criteria were further analyzed. Based on the currently available evidence, dysregulation of miRNAs associated with prognosis has been identified only in adenoid cystic carcinoma (AdCC) and mucoepidermoid carcinoma (MEC). Most studies originated from Asia and used qRT-PCR for miRNA assessment. Most researchers focused on the evaluation of selected candidate miRNAs, rather than conducting comprehensive miRNA profiling approaches in relation to prognosis. Several distinct dysregulated miRNAs were identified across the studies. Among them, miR-9, miR-21, miR-24 miR-29, miR-34c-5p, miR-125a-5p, miR-143, miR-145 and miR-205 were reported in more than one study and linked to clinicopathological advancement and a more aggressive disease course, thus leading to a poorer outcome. MiRNAs appear to represent promising prognostic biomarkers, either as independent predictors or in combination with established clinicopathological features. However, current evidence remains limited to selected histological subtypes and relatively small patient cohorts. Further well-designed studies with long-term follow-up are required. Moreover, comprehensive miRNA profiling with prognosis assessment is necessary before specific miRNAs could be reliably established as biomarkers and utilized in daily clinical practice.

## 1. Introduction

Salivary gland cancers (SGCs) are rare neoplasms, comprising approximately 8.5% of all head and neck malignancies. Most cases originate in the parotid gland and exhibit a slight male predominance [1]. According to the World Health Organization (WHO), more than 20 distinct histopathological subtypes have been identified [2,3]. The considerable morphological diversity among these subtypes presents significant challenges in accurate differential diagnosis [4,5,6] and might lead to a delay in management including proper treatment. Mucoepidermoid carcinoma (MEC) and adenoid cystic carcinoma (AdCC) occur most commonly. SGCs are frequently characterized by an aggressive clinical course and poor prognosis, which vary according to histopathological type, particularly in cases with locoregional recurrence or distant metastases [1,7,8,9,10]. In AdCC, recurrences are described in up to 15–85% of cases during long-term follow-up [7,11,12]. Currently, clinical and histopathological features, such as perineural invasion, vascular invasion, tumour grade and stage, and solid growth pattern in AdCC, are recognized as factors associated with relapse or metastasis, indicating an unfavourable outcome [7,8,9,13,14,15,16,17,18,19,20]. Earlier reports demonstrated a significant impact of these factors on survival [12,21]. Consistent with these findings, in our previous research, patients who developed recurrence or metastasis (poor oncologic outcome group) had significantly reduced survival in comparison to those in the favourable outcome group (disease-free survival (DFS)—2.4 vs. 8.3 years; overall survival (OS)—5.4 vs. 8.3 years; respectively) [22]. Surgical resection remains the gold standard for the treatment of SGCs, followed by adjuvant radiotherapy or cisplatin-based chemoradiotherapy, depending on clinical and pathological indications [23,24].

Although genetically specific alterations might turn out to be pivotal in differential diagnosis [2], their role in prediction might not be significant [25]. However, further studies to establish the molecular landscape in SGCs are needed, especially those focusing on molecules that might play a role as biomarkers. The identification of both diagnostic and predictive biomarkers in SGCs is critically needed.

MicroRNAs (MiRNAs) are small non-coding RNA molecules that regulate post-transcriptional gene expression [26,27]. The critical involvement of miRNAs in the regulation of diverse biological processes has been increasingly highlighted across various diseases, including cancer. There are many different miRNAs that might play a role as oncogenes (oncomiRs) or tumour suppressors. Dysregulated miRNAs are involved in numerous key biological processes, including cell proliferation and differentiation, apoptosis, tumorigenesis, epithelial–mesenchymal transition (EMT), metastasis, and angiogenesis [26,27,28,29]. Various abnormalities in miRNA expression have been reported across numerous carcinoma types and, in many cases, were associated with poor prognosis [29,30,31,32]. A variety of miRNAs target pivotal genes that affect numerous signalling pathways involved in carcinogenesis, such as *PTEN*, *RPS7*, *LIMCH1*, *MAPK1*, *MAP2K1*, *FOXC1*, *PDCD4*, *ESR1*, *ZEB2*, and *BTBD1* [33,34]. Furthermore, studies have shown that alterations in miRNA expression influence treatment outcomes. For instance, the downregulation of miR-29 has been associated with resistance to cisplatin, which is the main systemic agent used in head and neck cancers [30]. Moreover, various miRNAs across numerous cancer types have been established as both diagnostic and prognostic markers [28,29]. These findings suggest that miRNAs hold significant promise not only as biomarkers for diagnosis and prognosis but also as potential therapeutic targets.

In contrast, in the case of head and neck cancers (HNCs), especially SGCs, miRNA analysis has not been comprehensively performed. The most common miRNA abnormalities in SGCs have been described predominantly in AdCC and MEC.

To date, few systematic reviews have examined miRNAs in salivary gland malignancies. However, most of these reviews focus either on specific histopathological types or provide general information, while no comprehensive systematic review focusing on patient prognosis in SGCs has been conducted to date.

The objective of this study was to assess the dysregulation of microRNA expression across various subtypes of SGCs, with a particular focus on its potential role in prognosis.

## 2. Methods

### 2.1. Data Source and Search Strategy

#### 2.1.1. Protocol and Registration

The study was conducted in accordance with the PRISMA 2020 statement [35]. The study protocol was pre-registered in PROSPERO (CRD420251231827). Four databases (PubMed/MEDLINE, Embase, Web of Science and the Cochrane Library) were searched for studies published before 3 December 2025, using a combination of the following keywords: (salivary gland malignancies OR salivary gland cancers OR salivary gland carcinoma OR salivary gland tumours OR mucoepidermoid carcinoma OR myoepithelial carcinoma OR salivary duct carcinoma OR epithelial-myoepithelial carcinoma OR carcinoma ex pleomorphic carcinoma OR adenocarcinoma OR acinic cell carcinoma OR adenoid cystic carcinoma OR secretory carcinoma OR polymorphous adenocarcinoma OR cribriform adenocarcinoma OR microsecretory adenocarcinoma OR basal cell adenocarcinoma OR mucinous adenocarcinoma OR clear cell carcinoma OR intraductal carcinoma) AND (miRNA OR microRNA) AND (prognosis OR outcome). Extracted records were imported into Rayyan.ai (Rayyan Systems, 2026) [36] for duplicate removal, which was additionally verified manually by J.P. Next, J.P. and A.R. independently screened the titles and abstracts of the retrieved articles and selected potentially relevant publications for full-text assessment. The reference lists of included studies and prior systematic reviews were also examined to identify additional eligible studies. Disagreements during the screening process were resolved through discussion between the co-authors.

#### 2.1.2. Selection Criteria

Eligible study designs included observational cohort and cross-sectional studies reporting microRNA dysregulation in primary malignant salivary gland tumours (the most common histological subtypes) that reported at least one outcome relevant to the review objectives.

Studies were excluded if only the abstract was available, if the publication was a review or other non-original work, if the material was not tissue, if the histopathological subtype was non-malignant or included other malignant tumours, if the study did not assess prognostic characteristics, if the study was conducted in vitro or in vivo, if it was comparison between different histopathologic subtypes of SGCs, or if the analysis was based solely on bioinformatics databases.

#### 2.1.3. Outcomes

We included studies that reported the following outcomes:

- Metastasis (metastasis vs. no metastasis), (lymph node or distant)
- Recurrence (recurrence vs. no recurrence)
- Growth pattern in AdCC (solid growth pattern vs. cribriform/tubular pattern)
- Tumour grade in MEC (high-grade vs. intermediate-grade or low-grade)
- Perineural invasion (perineural invasion vs. no perineural invasion)
- Tumour size/stage (measured in centimetres/T1–T2 vs. T3–T4)
- Localization (major/minor salivary glands)
- Survival outcomes (overall survival, disease-free survival, recurrence-free survival, and cumulative survival rate, cumulative proportion survival), measured in months

**Figure 1:**
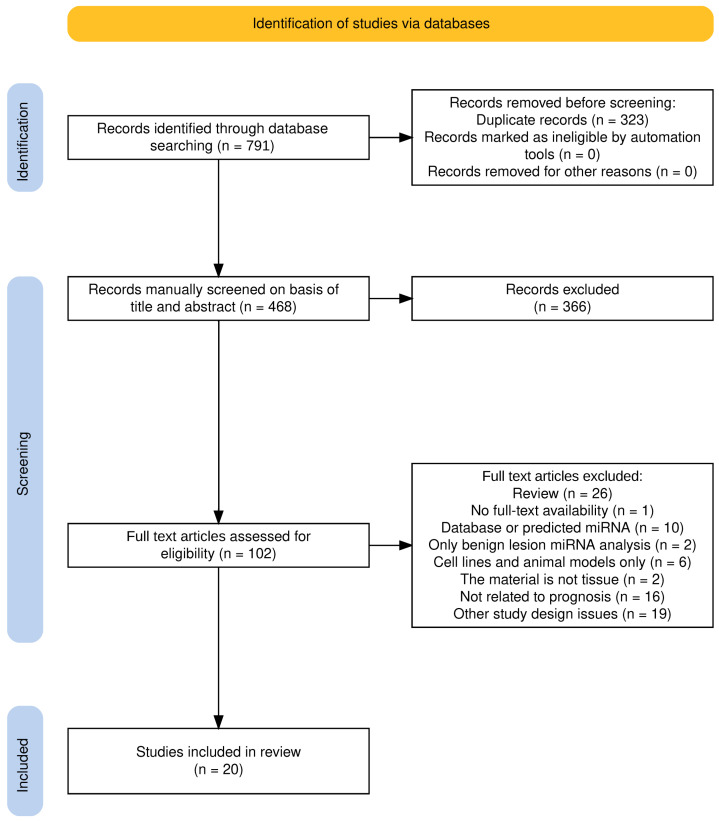
Flow diagram summary of item selection in the systematic review (PRISMA) [37].

The number of reports excluded at each stage is indicated, along with the reasons for exclusion, in the PRISMA flowchart (Figure 1).

#### 2.1.4. Data Extraction

Data were extracted from the studies that met the inclusion criteria into a Microsoft Excel file for further data synthesis and the evaluation of study quality.

The following data were extracted from each study: publication data (author’s name, year of publication, title), population characteristics (number of participants, age reported as mean ± standard deviation/median and interquartile range), tumour characteristics (histopathological recognition, sample source, stage), study design, details regarding the conduct of the study, number of miRNAs analyzed, miRNAs detection technique, dysregulation of the selected miRNA, available outcome data, and the results of statistical analyses. Proportions across studies were summarized using the median and interquartile range (IQR) as primary measures due to expected clinical and methodological heterogeneity. Additionally, sample-size-weighted means were calculated as descriptive secondary summaries reflecting the distribution among all included participants. Two authors independently evaluated the risk of bias in the included studies without blinding to authors or journals. Studies were assessed using the Quality in Prognosis Studies (QUIPS) tool [38]. The overall risk of bias was categorized as low, moderate, or high, based on domain-level judgments. Disagreements in bias assessment were resolved through discussion among the reviewers. Risk of bias assessments were used to inform the interpretation of findings but did not lead to study exclusion. Visualization of the bias scores was performed with the robvis tool (2025) [39]. Owing to substantial clinical, methodological, and outcome heterogeneity across the included studies—including differences in study design, biological models, miRNA targets, outcome definitions, and the reporting of effect estimates—a comprehensive quantitative meta-analysis was not considered methodologically appropriate. Therefore, the findings were synthesized narratively. Where feasible, results were structured and tabulated to allow a comparison of the direction and consistency of associations across studies.

## 3. Results

### 3.1. Study Selection

A total of 791 articles were obtained from PubMed/MEDLINE, Embase, Web of Science, and the Cochrane Library. A further 323 duplicate records were removed before screening. After title and abstract screening, 366 records were excluded due to a lack of relevance. A total of 102 articles were included in the full-text assessment, of which 26 were excluded because they were review papers. The reasons for the exclusion of 56 other studies are shown in Figure 1. After the full-text assessment, 20 articles were included in this systematic review [33,40,41,42,43,44,45,46,47,48,49,50,51,52,53,54,55,56,57,58]. The reference lists of all included articles were manually screened to identify additional relevant studies.

**Figure 2:**
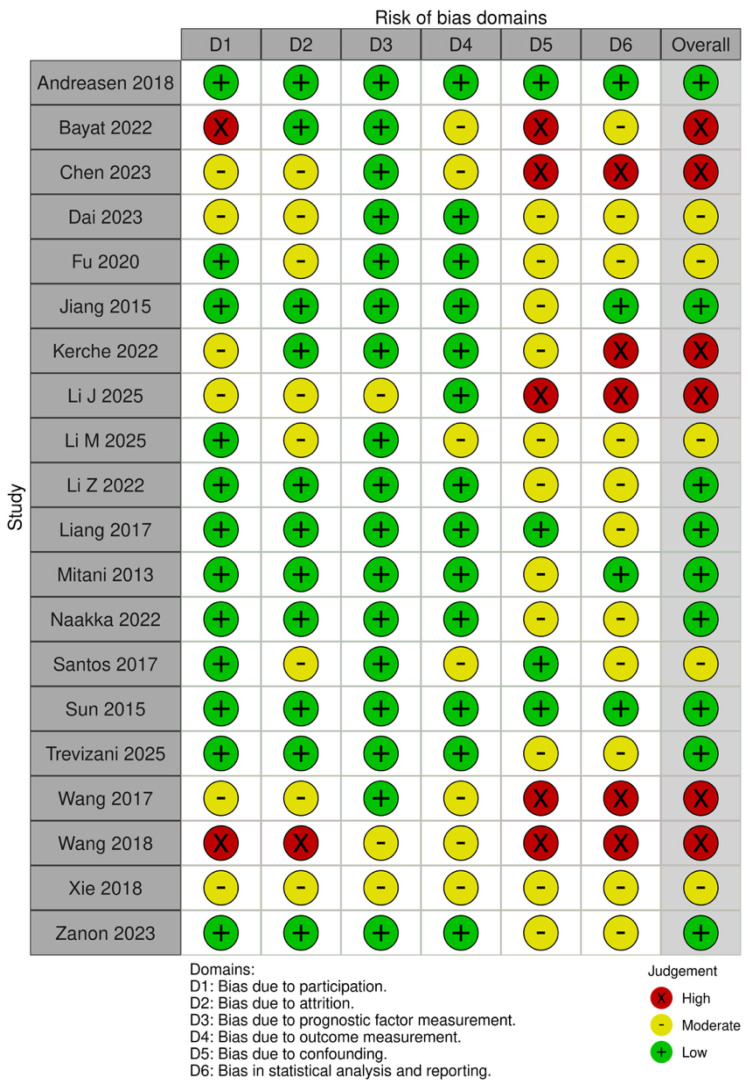
Risk of bias assessment using the QUIPS tool [33,40,41,42,43,44,45,46,47,48,49,50,51,52,53,54,55,56,57,58].

The risk of bias was assessed using the QUIPS tool across six domains. Overall, nine studies were judged to be at a low risk of bias, five at a moderate risk, and six at a high risk. A high risk of bias was assessed due to limited reporting on recruitment procedures, population representativeness, and variable reporting of losses to follow-up and the handling of missing data. Significant bias originated from incomplete adjustment for key prognostic variables. The details of the assessment are presented in Figure 2.

### 3.2. Study Characteristics

A total of 20 studies were included in this systematic review. The studies were published between 2013 and 2025, with the majority originating from Asia (65%; 12 from China, one from Iran), and 25% from Brazil. The main characteristics of the included studies are summarized in Table 1, which presents the results of miRNA analyses performed exclusively on human primary malignant tumours, including an assessment of patient prognosis.

Considerable heterogeneity was observed among the included studies. In most cases, clinical and demographic data were limited, particularly in studies that included additional in vitro and/or in vivo experimental components. Moreover, in most studies (approximately 75%), the analyses focused on individual miRNAs or small panels of selected miRNAs, whereas miRNA profiling approaches were less frequently applied.

In 16 studies, the histopathological recognition included AdCC [40,41,42,43,44,45,47,48,49,50,51,53,55,56,57,58]. Two studies analyzed only MEC tumours [33,54], while two studies contained both tumour types [46,52]. In 65% of the studies, tumours originated from major or minor salivary glands. Two studies analyzed tumours exclusively from the parotid gland, while in five studies localization was not specified in detail.

Regarding tissue preservation, FFPE samples were used in 7 studies (35%), while fresh-frozen tissue was analyzed in another 6 studies (30%). One study utilized both FFPE and fresh-frozen specimens, whereas 6 studies (30%) did not specify the method of tissue preservation.

MiRNA expression analysis was mostly performed using qRT-PCR, with prior miRNA profiling by microarray conducted in two studies. In situ hybridization (ISH) and fluorescence in situ hybridization (FISH) were employed either independently or alongside complementary analytical techniques in six studies. Additionally, the NanoString nCounter Technology and the Agilent SurePrint 8x60K platform were utilized in the studies conducted by Zanon et al. and Naakka et al., respectively [33,58]. Experimental assays were often not described in detail, particularly with regard to technical replicates. Only a few studies reported performing analyses in triplicate or duplicate [40,43,44,45,46,47,48,50,51,55,57]. U6 was used as an endogenous control in most studies using PCR-based methods [41,43,44,45,47,48,50,55,57], whereas Kerche et al. and Santos et al. utilized U48 [46,52].

### 3.3. Data Characteristics

Several miRNAs were reported in more than one study. MiR-21 was among the most frequently dysregulated miRNAs. Its upregulation in SGCs was associated with a shorter OS and DFS, as well as with distant metastasis and PNI (miR-21-5p), in three independent studies [40,45,58]. Similarly, dysregulated miR-9 expression was related to poorer survival, solid growth pattern and PNI in AdCC [46,51,58]. However, Santos et al. evaluated miRNAs related to mast cell activation and angiogenesis, including miR-9, and did not observe significant associations with patient prognosis [52]. MiR-205 demonstrated heterogeneous dysregulation across studies. It was reported as upregulated by Bayat et al. and Naakka et al. (miR-205-5p), where it was related to tumour size in AdCC and shorter OS in MEC, respectively [33,41]. Furthermore, Naakka et al. demonstrated a role for miR-205 in migration and invasion in vitro. In contrast, Mitani et al. reported miR-205 downregulation, which was associated with a poorer survival and a solid growth pattern in AdCC [51]. A dual role was also observed for miR-155. Kerche et al. reported that upregulated miR-155 was related to a shorter OS in AdCC, whereas downregulated or non-altered miR-155 expression was observed in high-grade MEC [46].

The downregulation of miR-143 was described by Mitani et al. as being associated with shorter survival and a solid growth pattern in AdCC [51]. Xie et al. reported reduced miR-143-3p expression in AdCC cases with metastasis [57], whereas Zanon et al. reported overexpression of miR-143 in cases presenting with PNI [58]. Similarly, Mitani et al. identified downregulated miR-145 in AdCC cases with a solid growth pattern and a shorter survival [51]. In addition, Naakka et al. observed that downregulated miR-145-3p negatively affected OS in MEC [33].

Moreover, upregulated miR-21, miR-24, miR-29, miR-140, miR-143, miR-195 and other miRNAs presented in Table 1 were associated with perineural invasion [41,58]. Downregulation of miR-34c-5p, miR-409-3p, and miR-376a was observed in AdCC cases with a solid growth pattern in two independent studies [51,58]. Reduced miR-338-3p expression negatively impacted OS in MEC and was associated with lymph node metastasis in AdCC (similarly to miR-338-5p) [33,55]. Additionally, downregulated miR-582-5p was linked to distant metastasis and survival in AdCC, as well as to high-grade tumours in MEC [33,56].

The upregulation of miR-200c and the downregulation of miR-16, miR-24, miR-27a, miR-106a/b, miR-125a-5p, miR-191, miR-210, miR-324-3p, miR-339-3p, miR-374b-5p, miR-484, miR-590-5p, miR-671-3p, miR-744, and miR-886-3p/5p were linked to lymph node metastasis in MEC [46,54]. In addition, reduced expression of miR-24 and miR-27a was also associated with distant metastasis [54].

Additionally, elevated expression of miR-183-5p, miR-922, miR-103a-3p, and miR-21, as well as decreased expression of miR-582-5p, has been related to distant metastasis in AdCC [42,43,44,45,56]. In the studies conducted by Li et al., Liang et al., and Sun et al., reduced expression of miR-5191, miR-125a-5p and miR-320a was correlated with both lymph node involvement and distant metastasis [49,50,53]. Moreover, downregulated miR-143-3p was also associated with metastasis in AdCC [57].

**Figure 3:**
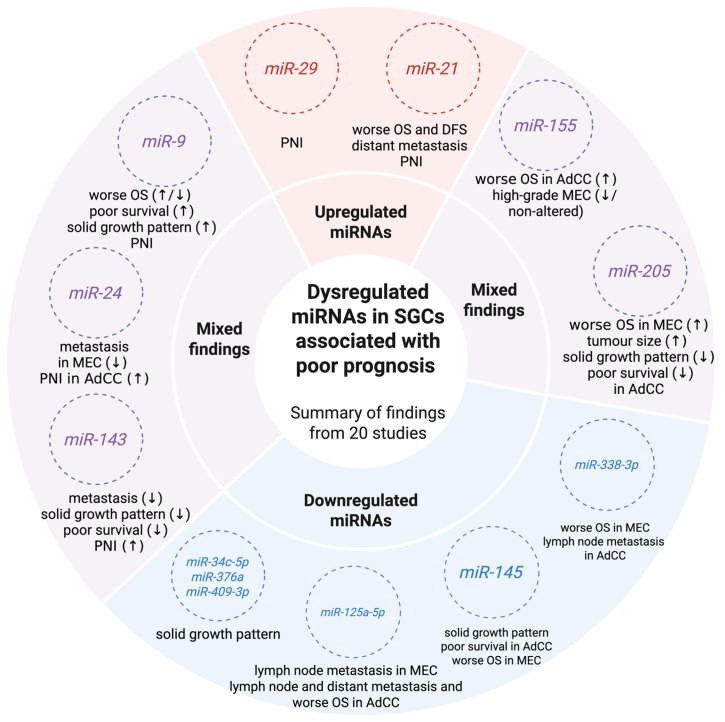
Summary of the most frequently reported dysregulated miRNAs associated with poor prognosis in salivary gland cancers. MiRNAs are grouped according to their predominant expression pattern (upregulated, downregulated, or mixed). In the mixed findings section, **↑**/**↓** indicates whether increased or decreased miRNA expression was associated with the reported adverse prognostic feature. Unless otherwise specified, the reported expression patterns refer to AdCC. Created in BioRender. Pikul, J. (2026) https://BioRender.com/dylzb1t.

A comprehensive overview of miRNA dysregulation and its associations with specific prognostic factors is presented in detail in Table 1, while recurrent miRNAs across studies or outcomes are graphically presented in Figure 3.

## 4. Discussion

The aim of this study was to systematically review the available evidence investigating the prognostic significance of miRNAs in SGCs, with the goal of improving clinical management and decision-making for these rare and heterogeneous tumours. Owing to substantial heterogeneity in study design, patient populations, and outcome measures, a meta-analysis was not feasible or methodologically appropriate. Nevertheless, this systematic review provides a comprehensive qualitative synthesis of the current evidence.

Most studies have focused on selected miRNAs, often previously reported as dysregulated in other malignancies, rather than performing comprehensive expression profiling. Despite this methodological limitation, certain miRNAs have been consistently identified across independent studies.

High expression of miR-21 was most frequently observed across studies and associated with poorer survival, distant metastasis, and perineural invasion in AdCC [40,45,58]. In vitro studies have shown the role of miR-21 in proliferation, apoptosis, migration, and invasion, and have emphasized its metastatic potential in human salivary adenoid cystic carcinoma (SACC) cell line [59,60]. Moreover, Wang et al. demonstrated in the highly metastatic potential SACC-LM cell line that the inhibition of miR-21 enhances the antitumor effects of simvastatin and may overcome drug resistance, suggesting a potential therapeutic strategy for clinical management [59]. Jiang et al. demonstrated using a multivariate Cox regression that miR-21 overexpression is an independent unfavourable predictor of survival in AdCC (HR 7.239; 95% CI: 1.081–48.460; *p* = 0.041) [45].

Several miRNAs may function either as oncomiRs or tumour suppressors in different cancers; nevertheless, their precise role has not yet been fully elucidated, even within a single cancer type (as shown in Table 2) [30,61,62,63,64,65,66]. The available literature suggests that the dual role of miRNAs may vary depending on the cancer type, histological subtype, tumour stage, tissue-specific target availability, and interactions with the tumour microenvironment, including immune cells. Because a single miRNA can simultaneously regulate multiple target mRNAs, its overall effect may differ depending on the biological context, contributing to the observed discrepancies in expression patterns [67,68,69]. Thus, the differing roles of individual miRNAs, even within the same tumour type, may reflect the biological complexity and context-dependent nature of miRNA-mediated regulatory networks rather than methodological inconsistencies alone [67].

In the present review, increased miR-155 expression was related to poorer overall survival in AdCC, whereas in the same study in MEC downregulated or non-altered miR-155 expression was observed in high-grade MEC [46]. Similarly, Mitani et al. showed that a lower expression of miR-205 was associated with a solid growth pattern and poorer survival, in contrast to Naakka et al. and Bayat et al., who linked elevated miR-205 expression to a shorter OS in MEC (miR-205-5p) and tumour size in AdCC, respectively [33,41,51]. Additionally, in the study conducted by Kerche et al., both upregulated and downregulated miR-9 expression influenced OS in AdCC, with 5-year survival probabilities of 25.3% and 0%, respectively [46].

The most consistently dysregulated miRNAs identified across the analyzed studies, along with their potential role as biomarkers in other solid malignancies, including HNCs are summarized in Table 2. Furthermore, particular attention should be given to miRNAs that have been independently reported in more than one study, as their recurrent identification may suggest stronger biological and clinical relevance.

For instance, the downregulated miR-34c-5p, which was associated with a solid growth pattern in AdCC by Mitani et al. [51] and Zanon et al. [58], is a well-known tumour suppressor that has been described in other solid tumours [70,71]. Re et al. revealed that decreased expression of miR-34c-5p in laryngeal SCC is an independent predictor of poor survival (shorter DFS and OS) and may be related to increased tumour aggressiveness, including metastasis and recurrence [70]. In gastric cancer, Wu et al. demonstrated that the downregulation of miR-34c-5p contributes to paclitaxel resistance in vitro, highlighting its potential role in chemosensitivity [71].

Liu et al. revealed that in pancreatic cancer, miR-429 was decreased in tumour tissues with PNI (*p* = 0.003) and a significantly lower expression was observed at stage III–IV in comparison with stage I–II [72]. These findings are consistent with the study conducted by Li et al., however, in their work, the association between miR-429 expression and PNI or metastasis in AdCC was demonstrated only in an experimental model [47]. Yang et al. demonstrated that downregulated miR-429 in the serum of patients with osteosarcoma corresponded significantly with tumour size (*p* < 0.001), clinical stage (*p* < 0.001), distant metastasis (*p* < 0.001), and survival (*p* = 0.041). Additionally, the researchers observed a similar correlation with the low expression of miR-143-3p [73]. MiR-143-3p and miR-145 have been reported to be downregulated in various malignancies, and their potential roles as diagnostic and prognostic markers have been highlighted [74,75,76]. In SGCs, Mitani et al. observed a reduced expression of miR-143 and miR-145 in tumours with a solid growth pattern and a worse survival in AdCC. Furthermore, Xie et al. and Zanon et al. linked miR-143-3p dysregulation to metastasis and PNI, respectively [51,57,58]. In parallel, Naakka et al. reported that decreased miR-145-3p expression in MEC correlated with a shorter OS [33].

In the comprehensive review by Hurník et al., the authors emphasize that studies evaluating miRNA expression correlation with PNI in HNSCC are relatively limited compared with other malignancies. Nevertheless, several miRNAs, including miR-21, miR-155, and miR-205 have been reported to be associated with PNI status in HNSCCs, suggesting their potential involvement in the molecular mechanisms underlying nerve invasion [77,78].

In SGCs, the elevated expression of miR-29 and miR-183-5p, as well as several additional miRNAs identified by Zanon et al. (Table 1) has been associated with PNI [41,42,58]. Conversely, the downregulation of miR-187, miR-361-5p, and miR-429 has also been linked to PNI but this has only been described in experimental models to date [47,79,80].

MiR-125a-5p is a well-known tumour suppressor whose dysregulation has been described in numerous cancers [81,82]. In a meta-analysis conducted by Ye et al. high miR-125a-5p expression was associated with a better OS in lung cancer (HR = 0.343, 95% CI: 0.228–0.517, *p* < 0.001) and gastric cancer (HR = 0.341, 95% CI: 0.160–0.725, *p* = 0.005). Additionally, its upregulated expression was significantly correlated with an early stage and negative lymph node metastasis in gastric cancer, as well as with better tumour differentiation across multiple cancer types [81]. In SGCs, decreased miR-125a-5p expression was associated with metastasis in both AdCC (lymph node and distant) and MEC (lymph node), as well as with worse OS in AdCC, where it was identified as an independent prognostic factor for poor survival [50,54].

**Table 2:** The most changed miRNAs in different solid tumours with emphasis on HNCs and their role in prognosis including therapeutic implications. ↑/↓ indicates increased/decreased miRNA expression or increased/decreased reported clinical outcome.

MiRNA	Role	Propose as a Prognostic Biomarker in Other Cancers	References
miRNA-9	oncomiR/tumour suppressor	↑ miR-9-3p vascular invasion, perineural invasion (sinonasal SCC) ↑ miR-9-5p lymph node metastasis, tumour growth, angiogenesis, invasion and influence on radiosensitivity (cervical cancer) ↓ OS (clear cell renal cell carcinoma) ↓ miR-9-5p regional recurrence (sinonasal SCC)	Kovaříková et al. [65], Wei et al. [83], Xie et al. [84]
↑ miRNA-21	oncomiR	HNC (including oral SCC) ↓ OS, ↓ DFS (HNC (including oral SCC); breast cancer, cholangiocarcinoma) PNI (HNC (including SCC); cholangiocarcinoma) advanced stage (HNC (including SCC) lymph node metastasis (cholangiocarcinoma)	Mariani et al. [85], Yu et al. [77], Šimić et al. [86], Hedbäck et al. [87] Huang et al. [88], Lü et al. [89]
miRNA-29	oncomiR/ tumour suppressor	↓ miR-29 cisplatin resistance in ovarian cancer; multiple clinicopathological associations reported in various cancers, sometimes with opposing effects	Yu et al. [90], Menon et al. [30], Kwon et al. [66]
miRNA-155	oncomiR/ tumour suppressor	↑ miR-155 lymph node/distant metastasis, advanced stage, ↓ overall survival, chemoresistance to cisplatin and 5-fluorouracil, affects immune response, thus immunotherapy (colorectal cancer) ↓ miR-155 progression and metastasis (colorectal cancer) ↓ OS (breast cancer) ↑ miR-155-5p regional recurrence, advanced stage (sinonasal SCC). ↓ survival, tumour grade, lymph node metastasis, paclitaxel resistance, invasive breast cancer vs. ductal carcinoma in situ (breast cancer) ↑ miR-155-3p reverses paclitaxel resistance (breast cancer)	Hussen et al. [62], Lü et al. [89], Kovaříková et al. [65], Li et al. [63], Zhang et al. [64]
miRNA-205	oncomiR/tumour suppressor	↑ miR-205 local recurrence, infiltrative growth pattern, PNI (cutaneous SCC). advanced stage, metastasis (ovarian cancer) ↓ miR-205-5p ↓ OS, distant metastasis, advanced stage (gastric cancer)	Cañueto et al. [91] He et al. [92], Zhang et al. [93], Qin et al. [61]

## 5. Future Perspectives

SGCs are characterized by aggressive behaviour and an unpredictable clinical course, with frequent local recurrences and distant metastases in most cases, even after initial adequate management. This is particularly evident in recurrent or metastatic (R/M) disease, where mortality remains high despite systemic therapies [1,12,21,22]. Consequently, there is an urgent need for reliable prognostic biomarkers that could identify patients at a higher risk of poor outcomes at an early stage of management.

Several studies have suggested that integrated miRNA expression signatures may provide additional prognostic value for the prediction of survival, recurrence, disease progression and tumour aggressiveness, particularly when combined with established clinicopathological variables. At the same time, it has been reported that conventional clinicopathological parameters do not always accurately reflect the tumour biological behaviour or correlate precisely with the clinical outcome [45,53,62,84,86,94,95,96,97]. In this context, miRNAs have emerged as promising indicators due to their role in tumour progression and invasion and their influence on treatment response. However, the number of studies performing comprehensive multivariable analyses remains limited.

For instance, Cañueto et al. demonstrated in cutaneous SCC that the dysregulation of miR-203 and miR-205 correlated better with prognosis than histopathological variables alone [91]. Similarly, Ganci et al. revealed that the upregulation of a four-miRNA signature (miR-21-3p, miR-21-5p, miR-96-5p, miR-429) in the peritumoral tissue and tumour of HNSCCs predicts local recurrence, independently of conventional clinical prognostic variables [98].

In the SGC studies mentioned above, Jiang et al. revealed that elevated miR-21 expression had a greater impact on the prognosis than the tumour clinical stage [45]. Moreover, in a multivariate Cox regression model, Sun et al. demonstrated that miR-320a could serve as a prognostic marker independent of TNM staging and highlighted its potential role as a therapeutic target [53]. Similarly, Liang et al. identified low miR-125a-5p expression as an independent prognostic factor for poor survival in AdCC (*p* = 0.003; HR = 0.5; 95% CI: 0.3–0.9) [50].

Notably, many of the miRNAs associated with a poor prognosis in SGCs have also been implicated in treatment response, including resistance to first-line chemotherapeutic agents such as cisplatin. Thus, the prevention or identification of treatment failure, especially in R/M cases, is an imperative need to provide patients with more personalized treatment options.

In a meta-analysis conducted by Jayaraj et al. numerous miRNAs were identified as being associated with chemoresistance to commonly used drugs in HNCs. These included the upregulation of miR-200c (with mixed patterns of dysregulation), miR-205, miR-21, miR-92a, miR-103, miR-20a, and miR-155, as well as the downregulation of miR-34a, miR-145, and miR-139-3p [99], which were also described as changed in the analyzed SGCs. Cisplatin resistance has also been reported in other solid malignancies, including colorectal cancer and ovarian cancer, where high miR-155 expression or a downregulated miR-29 were observed, respectively [62,90]. In contrast, we observed miR-29 upregulation in PNI cases, while its downregulation was found in SGCs compared with normal tissue [41,58]. Although miR-29 is predominantly described as a tumour suppressor, oncogenic and opposing functions have also been reported, even within the same cancer type, highlighting its context-dependent and dual biological roles [66]. Therefore, miRNA expression profiles should be interpreted with caution, taking into account the tumour type, tumour stage, biological context, and tumour heterogeneity.

However, well-designed study designs incorporating appropriate material and accounting for factors such as cohort characteristics are required to precisely elucidate the role of miRNAs in outcome prediction in SGCs.

Accurate prognostic models that stratify patients by risk could significantly improve treatment planning, surveillance strategies, and oncologic supervision.

Although numerous molecules have been proposed as potential biomarkers in SGCs, their clinical applicability remains limited [100], largely due to insufficient validation and lack of standardization.

Therefore, the identification and validation of novel prognostic biomarkers, either alone or in combination with established histopathological and clinical factors, remain a priority. MiRNAs represent promising candidates due to their stability and regulatory role in cancer-related pathways [26,28,101]. However, the successful translation of these approaches requires carefully designed studies, incorporating appropriate biological materials and accounting for relevant cohort characteristics and confounding factors [101,102,103], including geographic and ethnic diversity, as genetic ancestry and population-specific factors may influence miRNA expression profiles and their prognostic relevance [104,105]. Prospective, multicentre studies should therefore prioritize external validation in independent cohorts from different geographic regions, particularly non-Asian populations, to ensure robustness, validate findings, and elucidate potential population-specific differences in miRNA dysregulation and their prognostic impact, using standardized methodological approaches.

Future well-designed, multicentre studies with standardized methodologies are needed to establish and further validate the prognostic value of specific miRNAs. To improve the quality, reproducibility, and comparability of future research, we propose the development of standardized methodological and reporting guidelines for molecular analyses, including miRNA studies, in salivary gland cancers. Such recommendations should include comprehensive patient demographics (e.g., age, sex, ethnicity, relevant comorbidities, previous malignancies, and other potential confounding factors), detailed clinicopathological characteristics, including whether the analyzed lesion represents a primary tumour or recurrent disease, the histological subtype according to the WHO classification, the tumour site, the TNM stage, the tumour grade, perineural and lymphovascular invasion, surgical margin status, and other established prognostic factors, as well as complete information regarding treatment modalities, including the extent of surgery, neck dissection, and adjuvant radiotherapy and/or chemoradiotherapy. The follow-up should be sufficiently long, preferably exceeding five years, given the indolent clinical course of many salivary gland cancers and the occurrence of late local recurrences and distant metastases, with all oncological outcomes reported using clearly defined survival endpoints. Furthermore, future studies should provide a detailed description of the analyzed biological material, including the tissue type (fresh-frozen or FFPE), tissue processing and preservation methods, tumour cellularity, and RNA quality assessment. Whenever possible, parallel analyses of prospectively collected fresh-frozen specimens and FFPE-derived material should be encouraged to allow a direct comparison and assessment of the potential impact of fixation-related artefacts and pre-analytical variability on miRNA expression profiles and their prognostic interpretation. Molecular analyses should be reported using standardized methodologies, clearly specifying whether comprehensive miRNA profiling or targeted expression analysis was performed, the analytical platform used (e.g., qRT-PCR, microarray, or next-generation sequencing), normalization strategies, endogenous controls, quality control procedures, and validation methods. Finally, the findings should ideally be validated in independent multicentre cohorts and analyzed using multivariable statistical models adjusted for established clinicopathological prognostic factors. Such an approach may facilitate the identification of clinically meaningful and reproducible miRNA biomarkers, improve risk stratification, optimize treatment planning and oncologic surveillance, and ultimately improve patient outcomes.

## 6. Strengths and Limitations

This systematic review provides a comprehensive synthesis of miRNA dysregulation in the most commonly occurring primary SGCs, based exclusively on studies of human primary malignant tumours. This review highlights consistent miRNA dysregulations across independent studies, despite methodological heterogeneity, thereby strengthening the biological relevance of the identified miRNAs. By focusing on associations with clinicopathological features and patient outcomes rather than on descriptive expression changes alone, the review emphasizes the potential prognostic and translational value of miRNAs in these rare tumours.

Substantial heterogeneity among the included studies was identified. Notable differences were observed in the study design, type of analyzed material, miRNA detection methods, and the number of investigated miRNAs. Additionally, the studies varied considerably with respect to the number of samples, including patients’ characteristics. Importantly, key clinical data were frequently incomplete or insufficiently reported, and in several studies, long-term patient follow-up was unavailable or limited. This lack of comprehensive clinical annotation and survival data restricted the ability to perform robust clinicopathological correlations and survival analyses. Furthermore, the QUIPS assessment identified concerns mainly related to confounding and statistical analysis/reporting. These limitations may have influenced the magnitude and precision of the reported associations and should be considered when interpreting the findings. Since most of the existing research originates from Asian populations, patients from other regions may not fully benefit from the current evidence base, limiting the generalizability and global applicability of these findings.

## 7. Conclusions

The discovery of precise prognostic biomarkers for SGCs is imperative to optimize management and improve patient outcomes. However, this requires well-characterized cohorts with comprehensive clinical data, integrated miRNA profiling, and rigorous prognostic assessments to identify clinically meaningful biomarkers in these rare cancers. Current evidence suggests that specific miRNAs hold promise as prognostic biomarkers and therapeutic targets, offering new opportunities for personalized treatment and tailored patient care.

## Figures and Tables

**Table 1:** Summary of 20 included studies focusing on human primary SGCs based on patients’ prognosis correlation.

Author	Year	Country	Study Design	Type of Malignant SGC	Localization	Malignant Samples	Material	Main Technology Methods	Number of Analyzed MiRNA with Prognosis Assessment	Key Findings	Target Genes or Signalling Pathways
Andreasen et al. [40]	2018	Denmark	human observational study	AdCC	major and minor salivary glands	64—training cohort 120—validation cohort	FFPE	microarray qPCR (TaqMan)	microarray-numerous qPCR (32 highest-ranking miRNAs in survival analysis)	↑ miR-6835-3p * ↓ RFS ↓ miR-1180 ** ↓ miR-374c *** ↑ mir-4676 ** ↓OS ↑ **mir-21** *** ↑ mir-181a-2 *** ↑ mir-152 *** * results obtained by microarray in the training cohort ** results obtained by qPCR in the training cohort *** results obtained by qPCR in the validation cohort	NP
Bayat et al. [41]	2022	Iran	human tissue study	AdCC	major and minor salivary glands	15	FFPE	qRT-PCR (SYBR Green)	3	↑ **miR-29** PNI ↑ mir-93-5p histopathological grade **↑ miR-205** tumour size	*VEGF* PI3K/AKT and JAK/STAT pathways
Chen et al. [42]	2023	China	in vitro (cell line) study + human tissue study + animal study	AdCC	major and minor salivary glands	25	human tumour tissue NP	FISH	1	↑ miR-183-5p (at the leading edge) minor salivary glands, PNI, distant metastasis	*FAT-1/YAP1*
Dai et al. [43]	2023	China	human tissue study + in vitro (cell line) study + animal study	AdCC	major and minor salivary glands	23-FISH 11—qRT-PCR	23 FFPE/ 11 fresh frozen tissue (only cribriform/tubular type)	FISH qRT-PCR	1	↑ miR-922 solid growth pattern, local invasion *, distant metastasis * results obtained by FISH and qRT-PCR	*DEC2* *HIF-1*
Fu et al. [44]	2020	China	human tissue study+ in vitro (cell line) study + animal study	AdCC	major and minor salivary glands	52	human tumour tissue NP	qRT-PCR (SYBR Green)	1	↑ miR-103a-3p local regional recurrence, distant metastasis	*TPD52*
Jiang et al. [45]	2015	China	human tissue study+ in vitro (cell line) study	AdCC	major and minor salivary glands	35	fresh frozen tissue	qRT-PCR (TaqMan)	1	**↑ miR-21** distant metastasis, cumulative DFS **independent predictor of survival**	*PDCD4* *STAT3* and several other
Kerche et al. [46]	2022	Brazil	human tissue study	AdCC MEC	major and minor salivary glands	18 14	FFPE	qRT-PCR (TaqMan)	6	**↑/↓ miR-9** ↓ OS AdCC **↑ miR-155** ↓/non-altered miR-34a ↓ OS MEC ↓/non-altered miR-138 **↓/non-altered miR-155** high grade MEC ↑ miR-200c lymph node metastasis MEC	related to EMT
Li, J et al. [47]	2025	China	human tissue study+ in vitro (cell line) study + animal study	AdCC	major and minor salivary glands	26	fresh frozen tissue	qRT-PCR (SYBR Green)	1	↓ miR-429 T3–T4 vs. T1–T2 PNI and metastasis in cell lines	*ZEB1*
Li, M et al. [48]	2025	China	human tissue study + in vitro (cell line) study + animal study	AdCC	major and minor salivary glands	22	FFPE	FISH	1	↓ miR455-3p recurrence, ↓ OS, ↓ RFS, ↓ DMFS metastasis in vitro	*GNPNAT1*
Li, Z et al. [49]	2022	China	human tissue study+ in vitro (cell line) study+ animal study	AdCC	NP	30	fresh frozen tissue	qRT-PCR (SYBR Green)	1	↓ miR-5191 solid growth pattern, lymph node and distant metastasis, advanced stage, ↓OS/CSR	*NOTCH2*
Liang et al. [50]	2017	China	human tissue study + in vitro (cell line) study + animal study	AdCC	parotid glands	106	human tumour tissue NP	In situ hybridization qRT-PCR * (SYBR Green)	1	↓ **miR-125a-5p** lymph node metastasis, distant metastasis, ↓ OS independent prognostic factor for poor survival * 40 samples with metastasis or without metastasis	*p38/JNK/ERK*
Mitani et al. [51]	2013	USA	human observational study	AdCC	major and minor salivary glands	30—screening 30—validation	fresh frozen tissue	MiRNA array profiling qRT-PCR (SYBR Green) for validation (miR-455-3p, miR-455-5p, miR-375, miR-142-3p, miR-17, and miR-20a)	NP	↑ miR-17 ↓CPS ↑ miR-20a poor survival ↓: miR-143/**, miR-145/**, miR-205, miR-299-5p, miR-433, miR-452, miR-483-5p ↑: **miR-9**/**, miR-17, miR-20a, miR-92a, miR-501-5p, miR-545, miR-1909** solid growth pattern ↓: miR-22, **miR-34c-5p**, miR-127-3p/5p, miR-136/**, miR-143/**, **miR-145**, miR-154, **miR-205**, miR-329, **miR-376a** **/b/c, miR-377, miR-379, miR-381, miR-382, **miR-409-3p**, miR-410, miR-411, miR-432, miR-495, miR-654-3p ↑: **miR-9**/**, miR-17, miR-20a ↑ miR-let-7a tumour size, tumour stage, recurrence ↑ miR-150 lymph node, tumour stage numerous other miRNAs corelated with clinicopathological features not listed ** miRNA star strand	several
Naakka et al. [33]	2022	Brazil Finland	Human observational study + in vitro (cell line) study	MEC	major and minor salivary glands	25	fresh frozen tissue	Agilent SurePrint 8x60K platform	NP	↑ **miR-205-5p** ↓ OS ↑ miR-224-5p ↓ miR-139-3p ↓ **miR-145-3p** ↓ miR-148a-3p ↓ miR-186-5p ↓ **miR-338-3p** ↓ miR-363-3p ↓ miR-4324 ↓ **miR-582-5p** high-grade MEC ↓ miR-3125 ↓ miR- 4324 (miR-22 and **miR-205**- migration and invasion in vitro)	Several As follow: ERK/MAPK signalling, EIF2 signalling, PI3K/AKT
Santos et al. [52]	2017	Brazil	Human tissue study	AdCC MEC	major and minor salivary glands	11 9	FFPE	qRT-PCR (TaqMan)	6 (**miR-9**, miR-16, miR-17, miR-132, miR-195 and miR-221)	NS (histological pattern in AdCC, histological grade in MEC, tumour size, localization, PNI)	
Sun et al. [53]	2015	China	in vitro (cell line) study + animal study + human observational study	AdCC	NP	450 (from two Centres)	human tumour tissue NP	In situ hybridization	1	↓ miR-320a lymph node metastasis, distant metastasis ↓ CSR ↓ CMR **independent indicator for lung metastasis**	*ITGB3*
Trevizani et al. [54]	2025	Brazil	human tissue study	MEC	parotid glands	10	FFPE	qRT-PCR (TaqMan Array Human MicroRNA A Card)	377	lymph node metastasis ↓: miR-16, **miR-24**, **miR-27a**, miR-106a/b, **miR-125a-5p**, miR-191, miR-210, miR-324-3p, miR-339-3p, miR- 374b-5p, miR-484, miR-590-5p, miR-671-3p, miR-744, miR-886-3p/5p ↓ **miR-24**, ↓ **miR-27a** distant metastasis	
Wang et al. [55]	2018	China	in vitro (cell line) study + human tissue study	AdCC	NP	37	fresh frozen tissue	qRT-PCR (SYBR Green)	2	↓ **miR-338-3p**/5p lymph node metastasis	*LAMC2*
Wang et al. [56]	2017	China	human tissue study+ in vitro (cell line) study + animal study	AdCC	NP	110	human tumour tissue NP	In situ hybridization	1	↓ **miR-582-5p** distant metastasis, ↓CSR	*FOXC1*
Xie et al. [57]	2018	China	in vitro (cell line) studv + human tissue study + in vivo	AdCC	NP	102	human tumour tissue NP	qRT-PCR	1	↓ **miR-143-3p** metastasis	*ITGA6*
Zanon et al. [58]	2023	Brazil	human observational study	AdCC	major and minor salivary glands	19	FFPE	NanoString nCounter Technology	~800	solid growth pattern ↑: miR-146b-3p, miR-326, miR-370-5p, miR-491-3p, miR-516b-5p, miR-631, miR-770-5p, miR-1249-3p, miR-1297, miR-4461, miR-4755-5p ↓:miR-23b-3p, **miR-34c-5p**, miR-154-5p, miR-181d-3p, miR-320b, miR-323a-3p, miR-330-5p, **miR-376a-3p**, miR-382-5p, **miR-409-3p**, miR-433-3p, miR-455-5p, miR-487b-3p, miR-543, miR-766-5p, miR-758-3p + miR-411-3p, miR-3127-5p, miR-3190-3p, miR-5196-3p + miR-6732-3p perineural invasion (49 dysregulated miRNAs): miR-1-3p, **miR-9-5p**, miR-19b-3p, **miR-21-5p**, miR-23b-3p, **miR-24-3p**, miR-27b-3p, **miR-29a/c-3p**, miR-30a/e-5p, miR-92a-1-5p, miR-122-5p, miR-133a-3p, miR-140-3p, miR-141-3p, miR-142-3p, **miR-143-3p**, miR-146b-5p, miR-151a-3p, miR-195-5p, miR-203a-3p, miR-206, miR-208b-5p, miR-302d-3p, miR-424-5p, miR-494-3p, miR-497-5p, miR-510-5p, miR-517a-3p, miR-548aa + miR-548t-3p, miR-548z + miR-548h-3p, miR-613, miR-628-3p, miR-664b-3p, miR-1224-5p, miR-1253, miR-1257, miR-1275, miR-1276, miR-1322, miR-1323, miR-1972, miR-1976, miR-3065-5p, miR-3161, miR-3180-5p, miR-4531, miR-5001-5p	several; 13 molecular pathways, such as Notch, Wnt, Hedgehog, TGFB, Hippo, MAPK, STAT, P13K, RAS, chromatin modification, transcriptional regulation, DNA damage control, cell cycle, and apoptosis.

Replicated miRNA findings across independent studies/outcomes are bolded. ↑/↓ indicate divergent miRNA expression (up- or downregulation) and the direction of the associated outcome (e.g., ↓OS = worse OS).

## Data Availability

No new data were created or analyzed in this study. Data sharing is not applicable to this article.

## Abbreviations

The following abbreviations are used in this manuscript:

AdCC	adenoid cystic carcinoma
CPS	cumulative proportion survival
CSR	cumulative survival rate
DFS	disease-free survival
DMFS	distant metastasis-free survival
EMT	epithelial–mesenchymal transition
FFPE	formalin-fixed, paraffin-embedded
FISH	fluorescent in situ hybridization
HNCs	head and neck cancers
ISH	in situ hybridization
MEC	mucoepidermoid carcinoma
NP	not precise
NS	not significant
R/M	recurrent or metastatic
qRT-PCR	quantitative real-time PCR
QUIPS	Quality in Prognosis Studies
OS	overall survival
SCC	squamous cell carcinoma
SGCs	salivary gland cancers
WHO	World Health Organization
